# A Comparative Study of Freshwater Fish Burgers Made from Three Amazonian Species: Omega 3 Fortification and Sodium Reduction

**DOI:** 10.3390/foods13040565

**Published:** 2024-02-13

**Authors:** Alexander Iman, Juan D. Rios-Mera, Estefany Rengifo, Flavia Palomino, Rafael Vela-Paredes, Jessy Vásquez, Dora Enith García de Sotero, Erick Saldaña, Raúl Siche, Fernando Tello

**Affiliations:** 1 Departamento de Ingeniería de Alimentos, Facultad de Industrias Alimentarias, Universidad Nacional de la Amazonía Peruana, Iquitos 16002, Peru; alexander.iman@unapiquitos.edu.pe (A.I.); estefany.rengifo@unapiquitos.edu.pe (E.R.); fpal30@gmail.com (F.P.); rafael.vela.515@unapiquitos.edu.pe (R.V.-P.); jessy.vasquez@unapiquitos.edu.pe (J.V.); 2 Instituto de Investigación de Ciencia y Tecnología de Alimentos (ICTA), Universidad Nacional de Jaén, Jaén 06800, Peru; juan.rios@unj.edu.pe; 3 Facultad de Ingeniería Química, Universidad Nacional de la Amazonía Peruana, Iquitos 16002, Peru; dora.garcia@unapiquitos.edu.pe; 4 Sensory Analysis and Consumer Study Group, Escuela Profesional de Ingeniería Agroindustrial, Universidad Nacional de Moquegua, Moquegua 18001, Peru; esaldanav@unam.edu.pe; 5 Escuela de Ingeniería Agroindustrial, Facultad de Ciencias Agropecuarias, Universidad Nacional de Trujillo, Trujillo 13011, Peru; rsiche@unitru.edu.pe

**Keywords:** fish products, omega 3, sodium reduction, microencapsulation, oxidative stability

## Abstract

This study aimed to formulate burgers made from three Amazonian fish species: *pacu (Pyaractus brachypomus)*, *boquichico (Prochilodus nigricans)*, and *bujurqui (Chaetobranchus flavescens)*, focusing on sodium reduction and fortification with fish oil microparticles (FOM) rich in eicosapentaenoic acid (EPA) and docosahexaenoic acid (DHA). The proximal composition, sodium and calcium content, instrumental texture profile, fatty acid profile, sensory profile, and overall liking were evaluated. Differences in proximal composition and fatty acid profiles between the fillets were reflected in the burgers. Fortification with FOM increased EPA and DHA in the burgers; thus, they can be considered “high in omega-3 fatty acids” and reduced the n-6/n-3 ratio below 4. There were sensory attributes that could be related to lipid oxidation but reduced overall liking for less than 10% of consumers. Nevertheless, certain sensory attributes (grilled, characteristic, aromatic, tasty, tender, and juicy) had a positive impact on the overall liking of more than 20% of consumers, yielding adequate scores (between 5.60 and 5.71) on the 9-point hedonic scale. The production process must be optimized by knowing the fish fillet quality in depth, improving the FOM and burgers’ oxidative stability, and achieving an adequate sensory and hedonic profile by employing consumers’ vocabulary to characterize new products.

## 1. Introduction

Fish is considered the primary protein source in the Amazonian region of Loreto, Peru due to the abundance and availability of hydrobiological resources accessible to the entire population [1]. Consequently, Loreto has the highest per capita fish consumption in Peru, which is 4.5-fold (55.4 kg) more than the national average (16.2 kg) [2]. Typically, Amazonian freshwater fish species are consumed either fresh or dried and salted. However, the region’s tropical climate creates a spoilage-promoting environment, which might alter its nutritional quality, causing financial loss and severe health consequences [3].

To counteract these negative issues, it is crucial to implement strategies that will preserve the quality and safety of fish by transforming it into value-added products that meet consumer preferences. Within the Western diet, the fast-food sector offers highly appealing options, with burgers made with red meat being the most preferred and consumed. However, increased consumption of these products is generally associated with metabolic syndrome diseases such as cardiovascular diseases and cancer [4,5]. 

In this framework, using fish fillets may be a healthier alternative to produce innovative and nutritionally balanced burgers, as consumers perceive fish as a source of omega-3 fatty acids [6]. Among the most cultivated species in Loreto are *pacu* (*Pyaractus brachypomus*) and *boquichico* (*Prochilodus nigricans*), which are the third and fourth most cultivated species because locals highly accept them and are easily adapted to culture conditions [7]. However, in the capture of species of great commercial value, other species are caught unintentionally, as is the case of *bujurqui* (*Chaetobranchus flavescens*) [8].

Although fish from the Amazon can be an alternative for the production of burgers, it is essential to consider the nutritional profile of the fillets, mainly in levels of long-chain polyunsaturated fatty acids (PUFAs), such as the omega 3 docosahexaenoic acid (DHA) and eicosapentaenoic acid (EPA), whose health benefits have been well documented [9]. Freshwater fish generally contain fewer omega 3 PUFAs compared to marine species [10], which suggests that the use of Amazonian fish fillets would not be enough to achieve PUFA levels recommended by the health agencies; thus, the fortification of the food product would be necessary. The use of fish oil obtained from marine sources is a common practice for fortifying foods with long-chain omega 3 PUFAs, but direct incorporation of fish oils into a food matrix is a significant challenge due to their susceptibility to oxidation [11]. Hence, microencapsulation has become one of the most recommended technologies to protect sensitive compounds like PUFAs, allowing for their safe incorporation into foods [12]

In addition to the incorporation of omega-3, sodium reduction is another factor to consider in producing even healthier burgers. Our research group has recently reported that reducing sodium chloride in fish-based burgers is possible by direct reduction and subsequent substitution with calcium chloride [13]. The simultaneous strategy of reducing sodium and incorporating PUFAs into burgers could result in a highly healthy product; however, in this line of research, there are only a few reports on beef-based burgers [14,15]. Therefore, to determine the effectiveness of this strategy, it is necessary to apply it to other food matrices that also require reformulation towards a healthier profile. In this study, we evaluated for the first time the use of Amazonian fish species (*pacu*, *boquichico*, and *bujurqui*) to produce burgers under the approach of sodium reduction and fortification with PUFAs. Furthermore, they are species that do not receive industrial added value; thus, the research results could awaken the interest of academia, industry, and society for the development and consumption of healthier burgers in the Amazon region.

The objective of this study was to compare three Amazonian fish species for the development of burgers reduced in sodium and fortified with fish oil microparticles rich in EPA and DHA on the physicochemical composition, cooking losses, instrumental texture, fatty acid composition, oxidative stability, sensory profile, and consumer liking.

## 2. Materials and Methods

### 2.1. Materials

The following materials were used: fish oil (FO) from menhaden (Sigma Aldrich, St. Louis, MO, USA), type A gelatin (244 bloom Gelita South America, SP, Brazil), Arabic gum (Sigma-Aldrich, Saint-Quentin Fallavier, France), transglutaminase (TG) Activa TG-S^®^ (Ajinomoto, Itasca, IL, USA), oregano powder (Badia, Lima, Peru), and sodium erythorbate (Frutarom, Lima, Peru). *Pacu*, *boquichico*, and *burjuqui* fish were supplied by the fish farm “Fundo Tony” (Iquitos, Peru). The fish were filleted, vacuum-packed, and stored at −18 °C for less than one week. Pork backfat was used as a palatable ingredient in the burgers purchased in the local market (Iquitos, Peru). Spices (salt, monosodium glutamate, onion powder, garlic powder, and pepper powder) were purchased in the local market.

### 2.2. Fish Oil Microencapsulation

Fish oil microparticles (FOM) were produced by complex coacervation technique according to the methodology described by Tello et al. [16], with modifications. Two 100 mL gelatin type A and Arabic gum solutions were prepared in a 1:1 ratio with 2.5 mL/100 mL solutions. They were placed in a 50 °C water bath for 60 min. The gelatin solution was emulsified with 2.5 g of fish oil (containing 37.84 ± 1.52 PUFA, 24.71 ± 0.97 MUFA and 34.58 ± 0.96 SFA g/100 g oil) using an Ultra Turrax (ISOLAB, Eschau, Germany) at 10,000 rpm for 3 min. The Arabic gum solution and 400 mL distilled water (50 °C) were added. The pH was adjusted at pH 4.0 using solutions of 0.5 and 2.5 mol/L HCl and 0.1 mol/L NaOH. Afterwards, the system’s temperature was gradually lowered from 50 °C to 10 °C in an ice bath, keeping it under slow and constant magnetic stirring. The microparticles were washed with distilled water (pH 4.0) and filtered through a 25 μm mesh sieve. For the cross-linking, moist microparticles were cross-linked with TG (30 units [U]/g of protein) for 6 h, at room temperature, under magnetic stirring. Then, the microparticles were washed and filtered. Finally, the microparticles were freeze-dried at −80 °C in a Virtis freeze-dryer (LyoQuest, Azbil Telstar Technologies, Barcelona, Spain) and stored in plastic bags.

### 2.3. Encapsulation Efficiency (EE), Microstructure, and Oxidative Stability of FOM

EE determination was performed by extracting the oil in the moist FOM using the Bligh and Dyer [17] method. Then, EE was calculated by applying Equation (1):

(1) $$EE\left(\%\right)=\frac{\frac{Extracted\;oil\;\left(g\right)}{Total\;solids\;\left(g\right)}\;}{\frac{Initial\;oil\;\left(g\right)}{Total\;solids\;\left(g\right)}\;}\times 100a$$

The morphology and microstructure of FOM were evaluated using an optical microscope (ZEISS–Primo Start, Ct. Livonia MI, USA) coupled to a digital camera controlled by the Zen program 2.3—Blue edition (Zen Imaging Software, Jena, Germany) and a scanning electron microscope (LEO 435 VP, Leo Electron Microscopy Ltd., Cambridge, UK) with a voltage acceleration of 20 kV.

The oxidative stability was evaluated in unencapsulated fish oil (U-FO) and FOM once a week for four weeks at 45 °C in a climate chamber (Climacell ECO 111, MMM Group, Munich, Germany) by measuring the peroxide production following the standard method IDF 74A:1991. The storage temperature was 45 °C to accelerate the oxidation process, as Tello et al. [16] reported. To perform the analysis, U-FO (0.01 g) was diluted with 4 mL of chloroform/methanol (7:3 *v*/*v*), and an aliquot of 200 µL was used for the reaction. In the case of FOM, the oil was previously extracted following the Bligh and Dyer [17] method. Then, 200 µL of the extracted oil was added to 9.6 mL of a chloroform/methanol (7:3 *v*/*v*) solution. To assess color formation, 50 µL of an iron (II) chloride solution and 50 µL of 3.94 mol/L ammonium thiocyanate were added. The sample was agitated and left in the dark for 5 min; the absorbance was measured at 500 nm with a spectrophotometer (Thermo Scientific, UV–Visible Spectrophotometer, Genesys 150, Madison, WI, USA) in triplicate. The quantity of peroxides produced was determined using a standard curve for Fe^3+^, with concentrations varying from 1 to 20 µg [18,19].

### 2.4. Burger Preparation

The burgers were produced according to the methodology described by Saavedra et al. [13] with modifications. Three burger treatments, each corresponding to the three fish species used *(pacu*, *boquichico*, and *bujurqui*) were produced and replicated in three independent processes at different days. In each process were produced 52 burgers per treatment. For sodium reduction, 50% of NaCl content was substituted by CaCl_2_.

The following ingredients were used in the formulation: 70.0 g/100 g fish fillets, 17.0 g/100 g pork backfat, 7.25 g/100 g cold water, 3 g/100 g FOM, 0.375 g/100 g NaCl, 0.375 g/100 g CaCl_2_, 0.40 g/100 g garlic powder, 0.40 g/100 g onion powder, 0.40 g/100 g pepper powder, 0.40 g/100 g oregano powder, 0.39 g/100 g monosodium glutamate, and 0.01 g/100 g sodium erythorbate.

For processing, the fish fillets and pork backfat were minced separately in a meat grinder (0.8 cm disc) (model W82U5, Talsa, Chirivela, Valencia, Spain). Then, all the ingredients, including FOM, were mixed manually for 5 min and pressed into a burger mold of 100 g weight, 10 cm diameter, and 1 cm height. Finally, the burgers were vacuum-packed (Model, Boxer 35, Henkelman, ’s-Hertogenbosch, North Brabant, The Netherlands) and stored at −18 °C for subsequent analysis in the next 15 days of storage, except the TBARS analysis, which was performed within 8 weeks.

For texture profile, yield properties, and sensory analysis, the burgers were cooked using an electrical grill at 150 °C until the internal temperature of the burgers reached 75 °C. The temperature was measured using a digital thermometer with a stainless-steel sensor probe (model WT-1, Walfront, Lewes, DE, USA). For texture profile and yield properties, the samples were cooled at 25 °C and 45 °C for sensory analysis.

### 2.5. Proximate Analysis, Sodium, and Calcium Content

The proximate composition: moisture (air-drying method, 950.46), protein (Kjeldahl method, 981.10), lipids (Soxhlet method, 960.39), and ash (incineration method, 920.53) of raw fish fillets and burgers were determined in triplicate following the methodologies described by AOAC [20]. The total carbohydrate content was calculated by difference. Moreover, the calcium (AOAC 929.07) [21] and sodium (AOAC 966.16) [22] content were evaluated in the burgers.

### 2.6. Cooking Losses

Cooking losses were measured in triplicate according to Equation (2):

(2) $$\%\;\mathrm{Cooking}\;\mathrm{losses}=\frac{\mathrm{Raw}\;\mathrm{weight}\;-\;\mathrm{Cooked}\;\mathrm{weight}}{\mathrm{Raw}\;\mathrm{weight}}\;\times \;100$$

### 2.7. Texture Profile Analysis (TPA)

TPA was measured using a TA-HD Plus texture analyzer (Stable Micro Systems, Godalming, UK). The TPA was performed with a 7.5 cm cylindrical plate probe on cylindrical samples of 2.5 cm diameter and 1 cm height. The samples were compressed to 50% of their original size at a constant speed of 20 cm/min (pre-test speed and post-test speed: 20 cm/min) [23,24]. The following parameters were recorded: (1) hardness (N), (2) springiness, (3) cohesiveness, and (4) chewiness (N) [5,25].

### 2.8. Fatty Acid Profile

The fatty acid profile of fish oil, fish fillets, and raw burgers was determined by methyl esterification, as described by Hartman and Lago [26], with adaptations based on the AOCS [27] Ce 1b-89 method. The lipids of fish fillets and burgers were extracted using the Bligh and Dyer [17] method. The fatty acid methyl esters (FAME) were quantified using a gas chromatograph (Shimadzu, Series 2010 Plus, Kyoto, Japan) equipped with a Restek-Wax column (30 m × 0.32 mm i.d. × 0.25 μm film thickness) coupled to a flame ionization detector (FID). The initial column temperature was 60 °C, increasing to 210 °C at 20 °C/min and remaining for 7 min, then the program reached 240 °C at 30 °C/min, staying for 15 min. The injector and detector temperatures were set at 250 °C. Hydrogen with a linear velocity of 21.0 cm/s was used as carrier gas. The injection volume was 1.0 μL in a split mode of 1/10. The FAMEs were classified according to their retention time compared to the corresponding benchmarks (FAME C8-C22, Sigma-Aldrich, St. Louis, MO, USA). The analysis was performed within 15 days after the burger processing, and the results were expressed as g fatty acids/100 g of sample.

### 2.9. Lipid Oxidation of Burgers

Lipid oxidation of raw burgers stored at −18 °C for eight weeks was determined in triplicate by measuring the thiobarbituric acid reactive substances (TBARS) using the AOCS official method Cd 19–90 [28], with modifications detailed by Patinho et al. [29]. The absorbance (532 nm) was read using a spectrophotometer (Thermo Scientific, UV–Visible Spectrophotometer, Genesys 150, Madison, WI, USA). TBARS values were calculated using a standard curve (0.6, 1.0, 2.5, 5.0, 10.0 µmol/L) of 1,1,3,3-tetraethoxypropane and expressed in mg of malonaldehyde (MDA)/kg of sample.

### 2.10. Microbiological Analysis

To ensure that the fish burgers were safe for human consumption, the regulations according to the sanitary standard that establishes the microbiological criteria of sanitary quality and safety for food and beverages for human consumption [30] were considered, which recommends the determination of Aerobic mesophilic, *Escherichia coli*, *Escherichia coli* O157:H7, *Staphylococcus aureus*, and *Salmonella* sp.

### 2.11. Sensory Evaluation

#### 2.11.1. Consumers

One hundred self-reported consumers of burgers (75% men and 25% women; 18–40 years) were recruited. The panel reported that 14% of participants consume burgers every 15 days, 21.2% consume them 1–3 times a week, 18% consume them once a month, and 47% consume them rarely. The participants signed informed consent approved by the Institutional Research Ethics Committee of the Universidad Nacional de la Amazonia Peruana—UNAP (protocol No. PI-007-11/04/22-CIEI-UNAP), approved on 11 April 2022.

#### 2.11.2. Procedure

The sensory test was carried out at the UNAP Sensory Evaluation Laboratory in sessions of approximately 15 min, following the methodology described by [31]. The consumers were accommodated in individual sensory booths under artificial white light, and burger samples (~10–15 g) were presented monadically on plates coded with three random numbers following a Williams Latin Square design [32]. The samples were kept in plastic containers with airtight lids to maintain the temperature at 45 °C for 15 min. First, consumers rated their overall liking using a 9-point hedonic scale ranging from 1 = extremely dislike to 9 = extremely like [33]. Then, consumers completed a check-all-that-apply (CATA) questionnaire, choosing the attributes that best describe the sensory profile of burgers [34]; a total of 20 terms were selected based on previous studies [14,15] related to the sensory characteristics of fish burgers and the effect of FOM produced by complex coacervation. Water and crackers were provided to consumers for palate cleansing between samples.

### 2.12. Data Analysis

Data (except TBARS and CATA questions) were analyzed by a mixed analysis of variance (ANOVA), considering treatments as a fixed effect and burger processing as a random effect. Tukey’s test was used for pairwise comparison. Both analyses were performed at 5% significance.

For the CATA questions, the frequency of the sensory terms was calculated by counting the number of consumers who used the words to describe each sample [35]. After that, a correspondence analysis (CA) was performed using the frequency rate of the terms considering the Chi-square distances [36]. A penalty analysis (PA) was performed using the consumer’s responses to determine the mean impact of sensory terms on the overall liking of burgers [15,37]. TBARS results were analyzed by a factorial design considering treatments, storage time (weeks), and the interaction as sources of variation. Pairwise comparisons were performed according to Tukey’s test at 5% significance.

XLSTAT 2015 (Addinsoft, New York, NY, USA) and R (R Core Team, 2017) [38] software were used for data analysis.

## 3. Results

### 3.1. FOM Characterization

The EE for FOM was 83.4%, higher than that reported by Habibi et al. [39] for fish oil microparticles without crosslinking (EE of 76.66%), but similar to the work of Tello et al. [16], who observed that the crosslinking of coacervated microparticles helped increase EE levels up to 85%. However, it is essential also to consider the role of wall materials, since according to Zhang et al. [40], it is common to obtain high EE values when using a mixture of Arabic gum and gelatin. Thus, microencapsulation by complex coacervation and crosslinking with TG may be an efficient method to encapsulate omega 3-rich fish oil.

**Figure 1:**
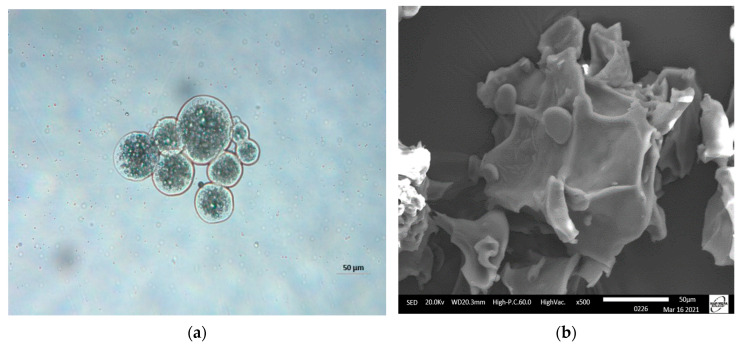
Micrographs of fish oil microparticles (FOM): (**a**) moist FOM (50 µm) (optical 798 microscopy) and (**b**) freeze-dried FOM (50 µm) (scanning electron microscopy).

Figure 1a shows the morphologies of moist FOM subjected to crosslinking with TG. It was observed that the microparticles had round shapes with thick walls. Likewise, they presented a multinuclear formation with the core material homogeneously distributed in the matrix. Similar morphologies were observed by Tello et al. [16] in crosslinked complex coacervates, which were used as reference in this study. Figure 1b shows that the microparticles lost their shapes and presented irregular structures with folds. This is common in freeze-dried complex coacervates and could be attributed to water loss during the freeze-drying process [41].

**Figure 2:**
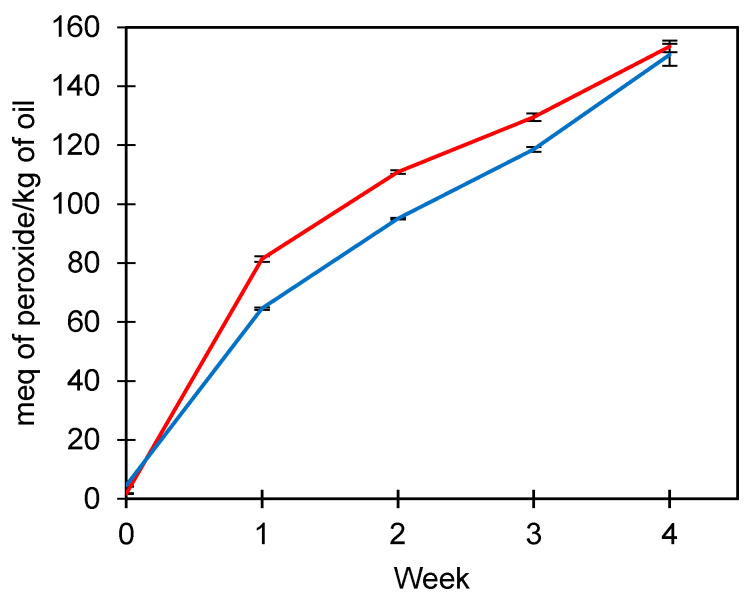
Peroxide index (meq of peroxide/kg of oil) of unencapsulated fish oil (red line) and fish oil microencapsulated (blue line) at 45 °C for four weeks. Average values ± standard deviation; three independent experiments (*n* = 3).

The peroxide index evaluated the oxidative stability of FOM under accelerated oxidation conditions at 45 °C (Figure 2). Initially, peroxides from FOM were slightly higher than U-FO (4.58 vs. 1.78 meq peroxide/kg oil), indicating that the manipulation of fish oil in the production of microparticles exerts an effect on the oxidation of fish oil. However, between weeks 1 and 4, the peroxide index of U-FO increased from 81.4 to 153.53 meq peroxide/kg of oil, while FOM obtained values between 64.58 and 150.71 meq peroxide/kg of oil. Therefore, the crosslinked microparticles exerted protection for fish oil during the time of peroxide index evaluation. Complex coacervates crosslinked with TG are known to be thermostable because of the formation of a network structure between glutamine and lysine [42].

### 3.2. Proximal Composition, Sodium, and Calcium Content

The proximal composition of fish fillets and burgers is shown in Table 1. For fish fillets, each of the species stood out in specific components of the proximal composition: *pacu* obtained the highest content of lipids and carbohydrates; *boquichico* also stood out in carbohydrates, as well as proteins and ashes; and *bujurqui* obtained the highest moisture value (*p* < 0.05). The protein and lipid content results for *pacu* differ from those of Murthy et al. [43], who reported 17 g/100 g and 1.19 g/100 g values for proteins and lipids, respectively. Similarly, Salas et al. [7] found protein and lipid contents of *boquichico* of 19.20 g/100 g and 3.47 g/100 g, respectively. The differences in lipid content may be because wild fish was used in those studies while we worked with farmed fish. According to Tanamati et al. [44], farmed fish have higher lipid content due to higher food availability and are usually confined in small tanks. Likewise, based on the lipid content, *bujurqui* can be classified as very low-fat (<2 g/100 g fat), whereas *pacu* and *boquichico* are medium-fat fish (4–8 g/100 g fat) [45]. It is essential to point out that the variations observed in the proximal composition of the fish species compared to other studies are typical due to factors like age, sex, environment, season, and diet composition [46].

Regarding the burgers, differences were observed (*p* < 0.05), except for ashes. The trend was similar to the proximal composition of the fillets: higher moisture content for *burjurqui* burgers, higher lipid and carbohydrate content in *pacu* burgers, and lower protein content for this treatment. The protein results are similar to those that Saavedra et al. [13] found in *pacu* burgers, where they observed protein values between 15.97 and 19.56 g/100 g. Still, lipid content was lower due to this study’s lower amount of animal fat. Reports about burger formulation using *boquichico* and *bujurqui* fillets are incipient.

Besides the burger fortification with FOM, the other approach was to reduce the NaCl content by incorporating CaCl_2_, based on the results of Saavedra et al. [13], who reduced up to 75% of NaCl in the *pacu* burger. In this sense, the burgers’ sodium and calcium contents were quantified, obtaining sodium values between 268.67 and 292.00 mg/100 g and calcium values between 244.61 and 288.33 mg/100 g. Previous studies have indicated that the sodium content in commercial burgers ranges from 400 to 1000 mg/100 g [47,48]. Therefore, the levels observed in this study are comparatively lower. Several reports have demonstrated that excessive sodium consumption can lead to hypertension, which is highly associated with cardiovascular and renal diseases [49]. As a prevention measure, the World Health Organization (WHO) recommends adults have a maximum sodium intake of 2 g/day (equivalent to 5 g of salt) [50]. Likewise, in line with the salt reduction targets for 2024 proposed by the Public Health Department of England, ideally, burgers should contain an average of 270 mg and a maximum of 335 mg of sodium per 100 g [51]. Consequently, the fish burgers formulated in this study successfully meet these recommended goals, potentially leading to significant public health benefits.

### 3.3. Texture Profile Analysis (TPA) and Cooking Losses

The TPA results showed no significant differences (*p* < 0.05) for springiness and cohesiveness, but hardness and chewiness were significantly lower in *boquichico* burgers (Table 2). The results are similar to those found by Presenza et al. [52], who reported hardness values ranging from 20.46 to 38.67 N and chewiness values between 7.85 to 18.80 N in *Colossoma macroporum* burgers elaborated with oatmeal and cassava starch. Meanwhile, other studies reported lower values for hardness: Atitallah et al. [53] reported 9.0 N for *Picochlorum* burgers, while Romero et al. [54] reported a maximum value of 20.57 N in *Pseudoplatystoma corruscans* burgers. For cooking losses, no differences (*p* > 0.05) were observed between treatments, which ranged from 33.43 to 34.13% (Table 2).

Differences in TPA and cooking losses are usually related to each other and the product’s proximal composition. For instance, lower fat and moisture contents and higher cooking losses result higher cooking losses, leadingto harderburgers with a firmer texture [23]. This trend was not observed in this study, and may be due to differences in the fish species used. The texture and the ability to bind water and fat in meat products are dependent on the degree of solubilization of meat proteins [31], which could be affected by intrinsic factors such as amino acid composition and sequence, protein structure, and protein type [55]. The differences in protein quality and functionality should be addressed in future studies to optimize the production of fish products of Amazonian species, for which there is very little information.

### 3.4. Fatty Acid Profile

The fatty acid profiles of U-FO, fish fillets, and burgers were determined (Table 3). In total, 22 fatty acids were identified; some, despite being present in the U-FO and fillets, were not detected in the burgers, and vice versa (lauric acid, cis-10 pentadecenoic acid, n-6 gamma-linolenic acid, and arachidic acid), which could have been lost during the processing of the microparticles and burgers or attributed to other ingredients of the burgers. As expected, the levels of EPA and DHA were high in FOU, contributing to the increase in PUFAs, the total n-3, and the low n-6/n-3 ratio. The *boquichico* fillets stood out in 16 fatty acids compared to the other fish species, including the presence of EPA and DHA. Likewise, it obtained the highest SFA values but compensated for the highest sum of total PUFA and n-3.

The fatty acid composition of fish meat is influenced by diet [56,57], where n-6/n-3 is considered a good index to evaluate its nutritional quality. The UK Department of Health [58] recommends a maximum n-6/n-3 ratio of 4.0 to achieve a balanced fatty acid intake. In this sense, it has been demonstrated that a higher n-6/n-3 ratio can promote the development of inflammatory and autoimmune disorders and a greater risk of cancer and cardiovascular disease [59]. On the contrary, a rise in n-3 PUFA levels (and a lower n-6/n-3 ratio) exerts suppressive effects [60]. In our case, *boquichico* fillets presented the most ideal n-6/n-3 ratio (0.76), followed by *bujurqui* fillets (2.25). Meanwhile, *pacu* fillets had the highest (13.33). Thus, consuming *boquichico* and *bujurqui* can be considered healthy and beneficial for human health. However, considering that *pacu* is one the most cultivated and consumed species in the region [7], the population would not be meeting the n-6/n-3 daily recommendation; thus, formulating products using its fillets and fortifying them with n-3 sources seems a great option.

Similar to the fatty acid profile of fish fillets, *boquichico* burgers stood out with 12 fatty acids, of which the most significant presence of EPA and DHA stands out. Likewise, it obtained the highest contents of SFA, MUFA, PUFA, n-3, n-6, and n-9, but the lowest n-6/n-3 ratio. It is essential to consider that fortification with FOM contributed to the presence of and increase in EPA and DHA in the burgers of the three species, which means that the microencapsulation process effectively carried these fatty acids into the burgers. Similar results were reported by Rios-Mera et al. [15], who observed that the fish oil microencapsulation by complex coacervation maintained the DHA in beef-based burgers compared to samples added with fish oil without encapsulation. Cross-linking with TG probably contributed positively to maintaining the fatty acids of interest in the fish oil, as observed by Tello et al. [16], who reported that TG concentrations above 30 U offer greater protection of the encapsulated oil. Regarding the n-6/n-3 ratio, fortification with FOM contributed to obtaining levels lower than 4.0, even in *pacu* burgers, indicating that the lipid profile of *pacu* fillets can be improved with the strategy proposed in this study.

On the other hand, according to the European Parliament [61], for a product to meet the requirements to obtain the nutritional claim of source orhigh in EPA/DHA, it should contain >40 mg and >80 mg of EPA+DHA, respectively. In our study, all the burgers fortified with FOM surpassed both recommended values. Therefore, the products developed in this study can be considered as “high in omega-3 fatty acids”. Besides representing a notable added value, nutrition claims are vital in improving the perceived image of processed products such as burgers [62].

### 3.5. Lipid Oxidation

**Figure 3:**
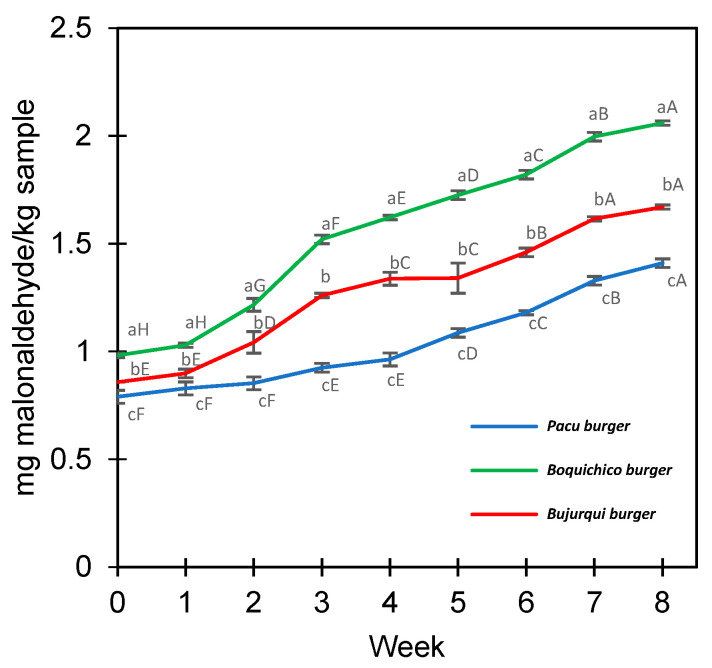
Lipid oxidation of fish burgers fortified with fish oil microencapsulated. Different letters between treatments (lower case) in each week and between weeks for the same treatment (upper case) represent a significant difference (*p* < 0.05) between the means obtained by Tukey’s test. Average values ± standard deviation; three independent experiments (*n* = 3).

The lipid oxidation of the raw fish burgers was measured during eight weeks at −18 °C by determining the levels of TBARS expressed in mg MDA/kg sample (Figure 3). All treatments increased TBARS during storage; from time 0 to week 8, *pacu* burgers ranged from 0.79 to 1.41 MDA/kg, *burjurqui* burgers from 0.86 to 1.67 mg MDA/kg, and *boquichico* burgers from 0.99 to 2.06 MDA/kg. The greater oxidation of *boquichico* burgers may be due to the greater presence of EPA/DHA (Table 3), fatty acids with multiple double bonds that make them more susceptible to oxidation [63]. Furthermore, it is necessary to know if the oxidation levels reached impact the sensory profile and overall liking of the product. Research has been conducted about determining the maximum threshold for TBARS values in meat products that do not cause any alteration in sensory characteristics and perception of oxidation by consumers. Devatkal et al. [64] and Zhang et al. [65] recommended a limit of 2 mg and 2.5 mg MDA/kg for beef products, respectively, meanwhile in other studies of oxidative stability involving fish (frozen, chilled, or stored in ice), values of up to 5 mg MDA/kg are considered acceptable [66,67]. In line with those studies, TBARS levels could be considered acceptable, and suggest that microencapsulation by complex coacervation effectively maintained the oxidative stability of fish oil. Previous studies reported that microencapsulation increased the oxidative stability of products containing fish oils [68,69,70]. However, to provide safe products for consumers in sensory analysis, the antioxidant sodium erythorbate was added to the burgers to prevent excessive oxidation. Hence, the contribution of this additive should not be disregarded.

Although the literature indicates sensorially acceptable TBARS limits, it is crucial to note, as highlighted by Jeong and Lee [71], that consumers’ perception and acceptability of food are influenced by cultural factors and their familiarity with the analyzed product. Therefore, the influence of the TBARS levels achieved (as well as the formulated products) on the sensory profile and overall liking of the products must be discussed under the conditions of this study.

### 3.6. Consumers’ Sensory Profile and Overall Liking

**Figure 4:**
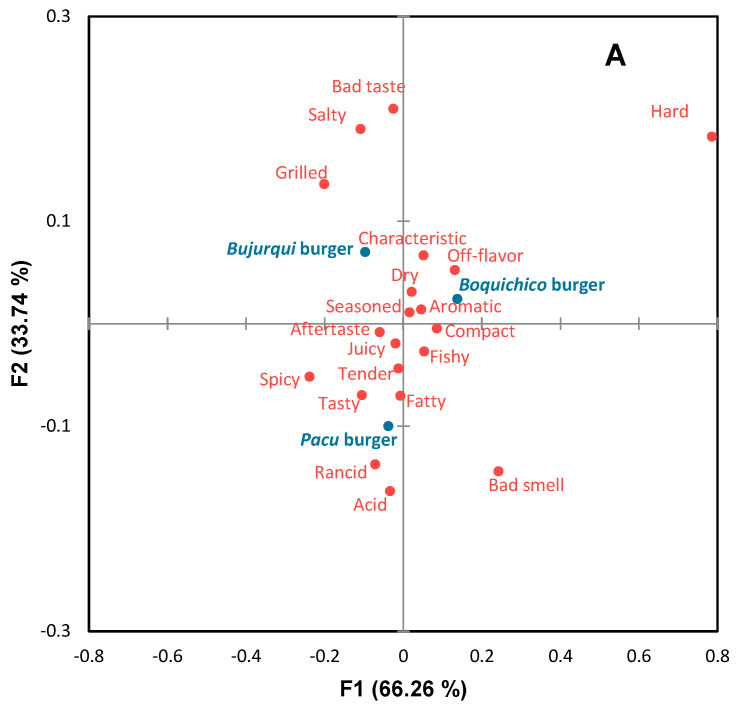

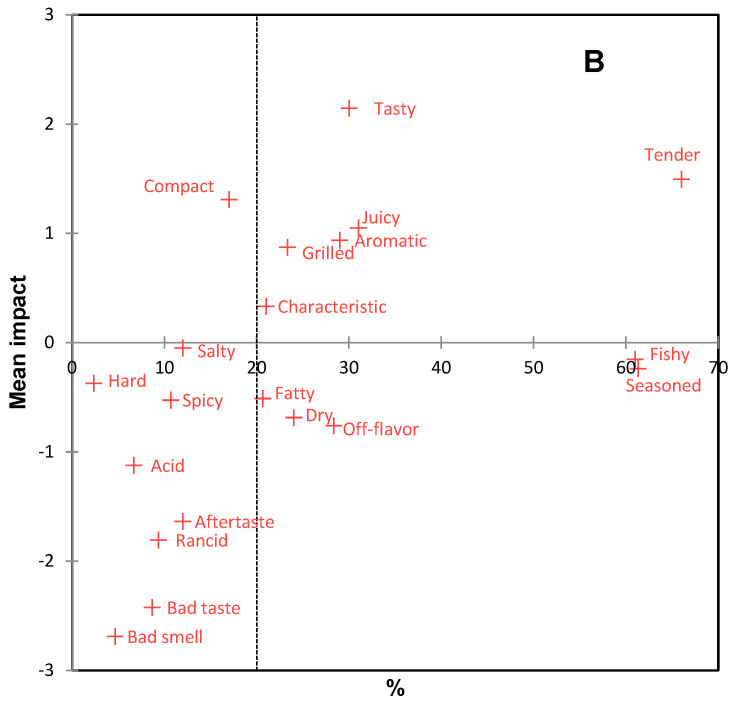
Correspondence analysis (CA) of (**A**) check-all-that-apply (CATA) questions of fish burgers fortified with fish oil microparticles, and (**B**) penalty analysis of the mean impact of sensory attributes on overall liking. Treatments are shown in blue and sensory attributes in red.

Before sensory analysis, the microbiological quality of the burgers was analyzed, and according to Peruvian regulations [30], they were considered safe for human consumption. The sensory profile of burgers is presented in the CA of Figure 4A. The first two dimensions accounted for 100% of the original variability of the data. Sensory differences were observed between the burgers, involving positive and negative attributes. According to Figure 4A, *bujurqui* burgers were characterized by *grilled*, *salty,* and *bad taste*; *boquichico* burgers had the presence of the attributes *characteristic off-flavor*, *dry*, *aromatic,* and *seasoned*; *pacu* burgers were characterized by *acid*, *rancid*, *tasty*, *fatty*, *spicy*, *tender*, *juicy* and *aftertaste*. The impact of these attributes on the overall liking of the burgers was determined by penalty analysis (Figure 4B), obtaining that the attributes *tender*, *tasty*, *juicy*, *aromatic*, *grilled*, and *characteristic* were essential to increase the overall liking of more than 20% of consumers, while the attributes *fishy*, *seasoned*, *fatty*, *dry*, and *off flavor* decreased overall liking for the same number of consumers. Likewise, other attributes were negative for less than 10% of consumers but greatly decreased overall liking, including *bad smell*, *bad taste*, *rancid*, *aftertaste*, and *acid*. The burgers of the three fish species had some of these attributes, which may be related to lipid oxidation. In this scenario, it could be indicated that the sensory results related to TBARS differ from the sensory acceptable limits of TBARS suggested in other studies [64,65,66,67]; however, as mentioned above, a minority of consumers used these negative attributes.

In the overall liking results, the samples obtained close averages: 5.64 for *pacu* burgers, 5.60 for *boquichico* burgers, and 5.71 for *bujurqui* burgers (*p* > 0.05). Saavedra et al. [13] received a higher overall liking rating (between 6.185 and 6.920) for *pacu* burgers reduced in salt and with the same ingredients as this study, except FOM. In this sense, fortification with FOM affected consumer acceptability, but not to the point of obtaining negative scores below half of the hedonic scale (4.5 note: “neither liked nor disliked”). The presence of sensory attributes related to lipid oxidation has likely caused a decrease in overall liking, and in this case, studying and defining the level of antioxidants in the encapsulation of fish oil by complex coacervation and the reformulated product, would be necessary, as recommended by Rios-Mera et al. [15].

On the other hand, as mentioned in the previous section (3.5 Lipid oxidation), cultural differences and the familiarity of consumers with the product analyzed [71] could explain the differences with the literature regarding the sensorially acceptable limits of TBARS, as well as the lack of consumer consensus to characterize each of the products. It is essential to mention that the products formulated in this study do not exist commercially. Hence, characterization may have been difficult for consumers during the evaluation. The use of techniques that involve consumers’ vocabulary in a given context can be helpful to characterize products; for example, Guàrdia et al. [72] sensorially described dry-cured ham using the free-choice profiling technique, which could give a better scope in the sensory characterization of newly formulated products, as in the case of this study.

Despite the contrasts in the sensory profile, it is possible to state that the overall liking of the burgers was positive for consumers. Therefore, transforming the Amazonian fish species used in this study into products such as burgers, reduced in sodium and fortified with FOM, can be a promising alternative for a highly healthy and nutritious food product in the Amazon region of Peru.

## 4. Conclusions

Burgers made from three fish species found in the Peruvian Amazon were produced, namely *pacu* (*Pyaractus brachypomus*), *boquichico* (*Prochilodus nigricans*), and *bujurqui* (*Chaetobranchus flavescens*). As freshwater fish typically have low levels of EPA and DHA, FOM produced through complex coacervation was added to increase the content of these fatty acids in the burgers for a more nutritious food source. The burgers were also reduced in sodium, obtaining adequate sodium levels according to recommendations of health agencies. Differences were observed in proximal composition, instrumental hardness and chewiness, fatty acid profile, lipid oxidation, sensory profile, and overall liking. Fortification with FOM was necessary for the presence of or increase in EPA and DHA in the burgers, and made them “high in omega-3 fatty acids” and low in n-6/n-3 ratio. Still, the sensory profile was variable for each burger, characterized by the presence of sensory attributes that positively and negatively impacted consumers’ overall liking. However, the overall liking scores were positive (between 5.60 and 5.71 on the 9-point hedonic scale), suggesting using *pacu*, *boquichico*, and *bujurqui* to produce burgers. To optimize the burger production process, future studies should focus on analyzing the properties of fish fillets, improving the oxidative stability of FOM and burgers under the antioxidant approach, and using sensory techniques that explore consumer opinions on new products.

## Figures and Tables

**Table 1:** Proximal composition of fish fillets and burgers elaborated with *pacu* (*Pyaractus brachypomus*), *boquichico* (*Prochilodus nigricans*) and *bujurqui* (*Chaetobranchus flavescens*), fortified with fish oil microparticles.

Proximal Composition (g/100 g)	Fish Fillets			Burgers		
	Pacu	Boquichico	Bujurqui	Pacu	Boquichico	Bujurqui
Moisture	75.52 ± 0.11 ^b^	74.00 ± 0.11 ^c^	76.55 ± 0.08 ^a^	61.04 ± 0.01 ^c^	61.81 ± 0.07 ^b^	65.36 ± 0.16 ^a^
Protein	15.42 ± 0.23 ^c^	17.84 ± 0.06 ^a^	17.15 ± 0.12 ^b^	15.27 ± 0.03 ^c^	17.54 ± 0.04 ^b^	17.77 ± 0.02 ^a^
Lipid	5.66 ± 0.11 ^a^	4.75 ± 0.04 ^b^	3.69 ± 0.07 ^c^	17.60 ± 0.06 ^a^	16.43 ± 0.09 ^b^	15.48 ± 0.08 ^c^
Ash	0.87 ± 0.01 ^b^	1.21 ± 0.01 ^a^	0.81 ± 0.02 ^c^	1.64 ± 0.03 ^a^	1.54 ± 0.08 ^a^	1.60 ± 0.06 ^a^
Carbohydrate	2.52 ± 0.18 ^a^	2.21 ± 0.14 ^a^	1.79 ± 0.20 ^b^	6.08 ± 0.02 ^a^	4.25 ± 0.20 ^b^	1.39 ± 0.08 ^c^

Average values ± standard deviation; three independent experiments (*n* = 3). Different letters on the same row represent a significant difference (*p* < 0.05) between fish fillets or fish burgers, according to Tukey’s test.

**Table 2:** Texture profile analysis and cooking losses of burgers elaborated with *pacu* (*Pyaractus brachypomus*), *boquichico* (*Prochilodus nigricans*), and *bujurqui* (*Chaetobranchus flavescens*), fortified with fish oil microparticles.

	Burgers		
	Pacu	Boquichico	Bujurqui
***Texture profile analysis***			
Hardness (N)	41.97 ± 5.49 ^a^	22.77 ± 3.18 ^b^	37.77 ± 3.77 ^ab^
Springiness	0.80 ± 0.04 ^a^	0.80 ± 0.02 ^a^	0.84 ± 0.04 ^a^
Cohesiveness	0.63 ± 0.04 ^a^	0.60 ± 0.01 ^a^	0.61 ± 0.01 ^a^
Chewiness (N)	21.11 ± 1.29 ^a^	11.90 ± 0.44 ^b^	19.27 ± 2.54 ^a^
***Cooking losses (%)***	33.43 ± 0.18 ^a^	34.06 ± 0.32 ^a^	34.13 ± 0.32 ^a^

Average values ± standard deviation; three independent experiments (*n* = 3). N: newtons. Different letters on the same row represent a significant difference (*p* < 0.05) between fish burgers according to Tukey’s test.

**Table 3:** Fatty acid profile of fish oil, fish fillets, and burgers elaborated with *pacu* (*Pyaractus brachypomus*), *boquichico* (*Prochilodus nigricans*), and *bujurqui* (*Chaetobranchus flavescens*), fortified with fish oil microparticles.

Fatty Acid (g/100 g)	Un-Encapsulated Fish Oil (U-FO)	Fish Fillets			Burgers		
		Pacu	Boquichico	Bujurqui	Pacu	Boquichico	Bujurqui
C12:0 Lauric acid	0.14 ± 0.01	N.D.	N.D.	N.D.	N.D.	N.D.	N.D.
C14:0 Myristic acid	9.86 ± 0.44	0.03 ± 0.00 ^b^	0.05 ± 0.00 ^a^	0.02 ± 0.00 ^c^	0.22 ± 0.01 ^c^	0.33 ± 0.01 ^a^	0.30 ± 0.02 ^b^
C14:1 Myristoleic acid	0.47 ± 0.03	0.00 ± 0.00 ^b^	0.02 ± 0.00 ^a^	0.00 ± 0.00 ^b^	0.00 ± 0.00 ^a^	0.00 ± 0.00 ^a^	0.01 ± 0.01 ^a^
C15:0 Pentadecanoic acid	0.78 ± 0.02	0.00 ± 0.00 ^b^	0.03 ± 0.00 ^a^	0.00 ± 0.00 ^b^	00.00 ± 0.00 ^b^	0.06 ± 0.01 ^a^	0.02 ± 0.02 ^b^
C15:1 Cis-10 pentadecenoic acid	0.06 ± 0.11	0.00 ± 0.00 ^a^	0.004 ± 0.01 ^a^	0.00 ± 0.00 ^a^	N.D.	N.D.	N.D.
C16:0 Palmitic acid	19.49 ± 0.45	0.57 ± 0.04 ^b^	0.68 ± 0.01 ^a^	0.24 ± 0.01 ^c^	2.86 ± 0.05 ^c^	3.77 ± 0.07 ^a^	3.33 ± 0.04 ^b^
C16:1 Palmitoleic acid	14.49 ± 0.21	0.08 ± 0.02 ^a^	0.10 ± 0.00 ^a^	0.03 ± 0.01 ^b^	0.58 ± 0.01 ^c^	0.96 ± 0.01 ^a^	0.66 ± 0.05 ^b^
C17:0 Margaric acid	0.79 ± 0.06	0.01 ± 0.00 ^b^	0.06 ± 0.00 ^a^	0.01 ± 0.01 ^b^	0.00 ^c^	0.12 ± 0.00 ^a^	0.08 ± 0.01 ^b^
C18:0 Stearic acid	3.66 ± 0.04	0.22 ± 0.02 ^a^	0.22 ± 0.01 ^a^	0.07 ± 0.01 ^b^	1.72 ± 0.03 ^b^	2.00 ± 0.04 ^a^	1.80 ± 0.04 ^b^
C18:1 n-9 Oleic acid	6.82 ± 1.11	0.80 ± 0.05 ^a^	0.28 ± 0.01 ^b^	0.19 ± 0.03 ^b^	4.73 ± 0.04 ^b^	5.22 ± 0.06 ^a^	4.89 ± 0.07 ^b^
C18:2 n-6 Linoleic acid	1.90 ± 0.01	0.34 ± 0.04 ^a^	0.15 ± 0.00 ^b^	0.12 ± 0.02 ^b^	0.70 ± 0.02 ^a^	0.62 ± 0.02 ^b^	0.58 ± 0.02 ^b^
C18:3 n-3 alpha-Linolenic acid	1.89 ± 0.04	0.02 ± 0.00 ^b^	0.15 ± 0.00 ^a^	0.02 ± 0.00 ^b^	0.22 ± 0.01 ^c^	0.37 ± 0.01 ^a^	0.24 ± 0.01 ^b^
C18:3 n-6 gamma-Linolenic acid	0.46 ± 0.04	N.D.	N.D.	N.D.	N.D.	N.D.	N.D.
C20:0 Arachidic acid	N.D.	N.D.	N.D.	N.D.	0.00 ± 0.00 ^a^	0.00 ± 0.00 ^a^	0.02 ± 0.01 ^a^
C20:1 Eicosenoic acid	1.18 ± 0.01	0.01 ± 0.00 ^b^	0.05 ± 0.00 ^a^	0.00 ± 0.00 ^c^	0.00 ± 0.00 ^b^	0.11 ± 0.01 ^a^	0.10 ± 0.01 ^a^
C20:2 Cis-11, 14-Eicosadienoic acid	0.24 ± 0.01	0.00 ± 0.00 ^b^	0.03 ± 0.00 ^a^	0.00 ± 0.00 ^b^	0.00 ± 0.00 ^a^	0.00 ± 0.00 ^a^	0.01 ± 0.02 ^a^
C20:3 n-3 Cis-11, 14, 17- Eicosatrienoic acid	0.25 ± 0.01	0.00 ± 0.00 ^b^	0.04 ± 0.00 ^a^	0.00 ± 0.00 ^b^	0.00 ± 0.00 ^a^	0.02 ± 0.03 ^a^	0.03 ± 0.01 ^a^
C20:3 n-6 Cis-8, 11, 14- Eicosatrienoic acid	0.33 ± 0.02	0.02 ± 0.00 ^b^	0.03 ± 0.00 ^a^	0.01 ± 0.00 ^b^	0.00 ± 0.00 ^a^	0.02 ± 0.04 ^a^	0.03 ± 0.00 ^a^
C20:4 n-6 Arachidonic acid	1.44 ± 0.05	0.04 ± 0.01 ^b^	0.10 ± 0.02 ^a^	0.04 ± 0.01 ^b^	0.17 ± 0.01 ^b^	0.31 ± 0.03 ^a^	0.12 ± 0.03 ^b^
C20:5 n-3 Eicosapentaenoic acid (EPA)	17.01 ± 0.61	0.00 ± 0.00 ^b^	0.06 ± 0.01 ^a^	0.00 ± 0.00 ^b^	0.17 ± 0.01 ^b^	0.36 ± 0.05 ^a^	0.21 ± 0.02 ^b^
C22:1 n-9 Erucic acid	1.69 ± 0.06	0.00 ± 0.00 ^b^	0.05 ± 0.00 ^a^	0.00 ± 0.00 ^b^	0.00 ± 0.00 ^a^	0.02 ± 0.04 ^a^	0.03 ± 0.00 ^a^
C22:6 n-3 Docosahexaenoic acid (DHA)	14.32 ± 0.89	0.01 ± 0.02 ^b^	0.12 ± 0.03 ^a^	0.06 ± 0.02 ^ba^	0.24 ± 0.01 ^b^	0.29 ± 0.02 ^a^	0.20 ± 0.02 ^b^
Total SFA	34.58 ± 0.96	0.82 ± 0.06 ^b^	1.05 ± 0.02 ^a^	0.34 ± 0.01 ^c^	4.80 ± 0.05 ^c^	6.29 ± 0.11 ^a^	5.55 ± 0.09 ^b^
Total MUFA	24.71 ± 0.97	0.89 ± 0.07 ^a^	0.51 ± 0.01 ^b^	0.22 ± 0.03 ^c^	5.31 ± 0.04 ^c^	6.31 ± 0.03 ^a^	5.69 ± 0.11 ^b^
Total PUFA	37.84 ± 1.52	0.43 ± 0.01 ^b^	0.69 ± 0.05 ^a^	0.26 ± 0.02 ^c^	1.49 ± 0.02 ^b^	1.97 ± 0.06 ^a^	1.42 ± 0.07 ^b^
Total n-3	33.48 ± 1.42	0.03 ± 0.02 ^b^	0.38 ± 0.03 ^a^	0.08 ± 0.02 ^b^	0.62 ± 0.01 ^b^	1.03 ± 0.06 ^a^	0.68 ± 0.05 ^b^
Total n-6	4.12 ± 0.10	0.40 ± 0.03 ^a^	0.29 ± 0.02 ^b^	0.18 ± 0.00 ^c^	0.86 ± 0.02 ^b^	0.95 ± 0.01 ^a^	0.73 ± 0.04 ^c^
Total n-9	8.51 ± 1.05	0.78 ± 0.07 ^a^	0.33 ± 0.01 ^b^	0.19 ± 0.03 ^c^	4.73 ± 0.04 ^c^	5.24 ± 0.03 ^a^	4.92 ± 0.07 ^b^
n-6/n-3 ratio	0.12 ± 0.00	13.33 ± 5.95 ^a^	0.76 ± 0.03 ^b^	2.25 ± 0.59 ^b^	1.39 ± 0.01 ^a^	0.92 ± 0.06 ^b^	1.08 ± 0.07 ^b^

Average values ± standard deviation; three independent experiments (*n* = 3). N.D.: not detected. Different letters on the same row represent a significant difference (*p* < 0.05) between fish fillets or fish burgers, according to Tukey’s test.

## Data Availability

Data is contained within the article.
